# EffectiveDB—updates and novel features for a better annotation of bacterial secreted proteins and Type III, IV, VI secretion systems

**DOI:** 10.1093/nar/gkv1269

**Published:** 2015-11-20

**Authors:** Valerie Eichinger, Thomas Nussbaumer, Alexander Platzer, Marc-André Jehl, Roland Arnold, Thomas Rattei

**Affiliations:** 1 Division of Computational System Biology, Department of Microbiology and Ecosystem Science, University of Vienna, 1090 Vienna, Austria; 2 Program in Genetics and Genome Biology, The Hospital for Sick Children, Toronto, Ontario M5G 1X8, Canada

## Abstract

Protein secretion systems play a key role in the interaction of bacteria and hosts. EffectiveDB (http://effectivedb.org) contains pre-calculated predictions of bacterial secreted proteins and of intact secretion systems. Here we describe a major update of the database, which was previously featured in the NAR Database Issue. EffectiveDB bundles various tools to recognize Type III secretion signals, conserved binding sites of Type III chaperones, Type IV secretion peptides, eukaryotic-like domains and subcellular targeting signals in the host. Beyond the analysis of arbitrary protein sequence collections, the new release of EffectiveDB also provides a ‘genome-mode’, in which protein sequences from nearly complete genomes or metagenomic bins can be screened for the presence of three important secretion systems (Type III, IV, VI). EffectiveDB contains pre-calculated predictions for currently 1677 bacterial genomes from the EggNOG 4.0 database and for additional bacterial genomes from NCBI RefSeq. The new, user-friendly and informative web portal offers a submission tool for running the EffectiveDB prediction tools on user-provided data.

## INTRODUCTION

Interactions between bacteria and eukaryotes are widespread in all ecosystems on earth and often lead to symbiotic relationships. The most prominent themes in current research are different types of human-microbe interactions, such as the interplay of human microbiomes with their host or human infections by bacterial pathogens. Understanding of bacterial interactions with other hosts, such as livestock animals and crop plants, are becoming crucial for sustaining nutrition and gaining renewable energy. Despite the major and fundamental progress in these fields due to novel molecular and computational methods, predictive modeling of complex host-microbe interactions is still limited (1). Among other challenges a better understanding of molecular mechanisms underlying microbe host interactions is needed.

Protein secretion is one of the major mechanisms for direct molecular interaction between bacteria and hosts and thus of fundamental importance. Several databases and web applications have been developed to search for proteins involved in bacterial protein secretion and for genomes encoding these. T346Hunter (2), SecReT4 (3) and SecReT6 (4) are specialized in the detection of bacterial secretion systems that are able to inject effector proteins directly into eukaryotic cells. T346Hunter (2) identifies core members of the Type III, IV and VI secretion systems by sequence similarity to conservation models. The tool provides the percentage of detected core components as utility for interpretation and estimating the potential functionality of secretion systems. SecReT4 (3) and SecReT6 (4) also focus on the known components of the secretion systems. The identified proteins can be obtained as a physical map. None of these three tools is able to make a binary decision whether a secretion system is intact or not. This decision, non-trivial due to the genetic flexibility and the limited knowledge about bacterial secretion systems (5), has to be made by the user. Considering the crucial role of presence and functionality of particular proteins for virulence mediated by protein secretion systems, as e.g. shown in (6), the role of computational predictions by generic models is to suggest and rank suitable candidates for further, more specific computational and experimental analysis.

Secreted protein sequences are mainly determined by their secretion signals. Among other tools, T3SEdb (7) and BEAN 2.0 (8) combine various approaches to predict Type III secreted proteins, such as machine learning approaches, domain annotation or by exploiting information of the conserved genomic context with secretion system core genes. Also additional features are included, such as chaperone-binding sites that facilitate the specific binding of chaperones to one or several effectors (9). Type IV secreted proteins can be recognized by their C-terminal signal sequence (10). Four types of distinctive features, amino acid composition, dipeptide composition, position-specific scoring matrix composition and auto covariance transformation of position-specific scoring matrix, were used to develop the classifier T4EffPred (11). T4SEpre (12) contains multiple models representing C-terminal sequential and position-specific amino acid compositions, possible motifs and structural features.

Predictions of intact protein secretion systems and secreted bacterial proteins would ideally be included in genome annotations, as available from primary DNA sequence archives (13). However, no standards for secretion-related annotations have been established so far. Also the microbial genome re-annotation initiative of the NCBI RefSeq team does not include a specialized method for protein secretion prediction (14). Therefore specialized secondary databases are needed to collect, structure and present secretion-related data in the context of a rapidly growing number of published microbial sequence records. Among other resources, PATRIC (15) is a sophisticated database covering diverse fields of pathogenicity. It offers, for example, predictions of antibiotic resistance and of virulence-related proteins. Nevertheless, comprehensive predictions of bacterial secreted proteins and of intact protein secretion systems, based on specialized and continuously updated prediction methods, were so far not available from a single publicly available resource.

We have therefore updated and expanded EffectiveDB (16), a database that provides pre-calculated predictions of bacterial secreted proteins as well as online tools for detecting effectors by their Sec-dependent and Type III secretion signals, and by predicting eukaryotic-like domains (ELD) which are likely to interact with host proteins. In order to create a comprehensive ‘one-stop-shop’ for analyzing genomes of host interacting microbes, we have improved the secretion prediction methodology of EffectiveDB and expanded its scope towards predictions of intact Type III, IV and VI secretion systems. Owing to the burst of sequenced genomes and metagenomes, and the constant increase in available experimental data, EffectiveDB assists biologists in the systematic analysis of microbial genomes and in short-listing putative genes for experimental studies of host microbe interactions.

## RESULTS

**Figure 1.**
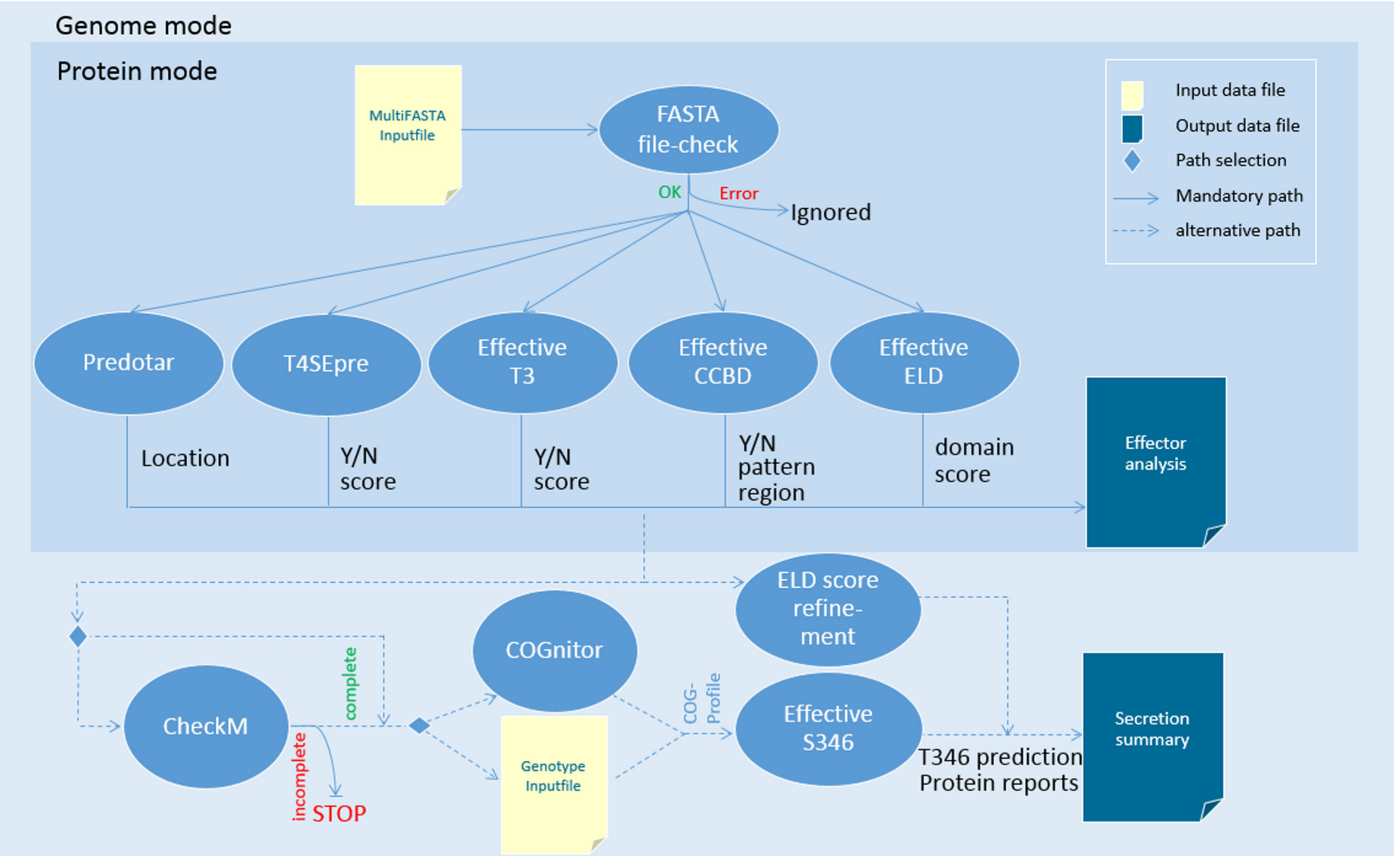
Integration of methods for the prediction of bacterial secreted proteins and intact secretion systems in EffectiveDB. The workflow depicts the protein and genome modes of EffectiveDB. In the protein mode any set of proteins can be analyzed. The genome mode extends the protein mode by enabling EffectiveS346. For proteins from (almost) complete genomes the orthologous groups are calculated or provided by the user. These are the input data for the prediction of intact Type III, IV and VI secretion system.

EffectiveDB is based on a suite of prediction programs for bacterial protein secretion. Pre-calculated predictions of secreted proteins and secretion systems resemble the core of EffectiveDB. While the previous release provided pre-calculations for only 1160 bacterial genomes, the updated version includes all 1677 bacterial genomes from EggNOG 4.0 (17) and for additional bacterial genomes from NCBI RefSeq (14). The EffectiveDB programs are also made available to the user for predictions of submitted protein sequences. According to the type of user input, the predictions are performed in ‘protein mode’, predicting secreted proteins for any collection of protein sequences, or in ‘genome mode’, also enabling the prediction of secretion systems and the discovery of novel ELD for proteins from nearly complete genomes (Figure 1). The EffectiveDB website also hosts documentation, software and data download, and supplementary information for all methods that have been specifically implemented for EffectiveDB.

### EffectiveS346 facilitates the prediction of intact secretion systems

The ‘EffectiveS346’ software has been implemented for EffectiveDB in order to predict secretion systems encoded in bacterial genomes and to provide a clear ‘yes/no’ prediction on whether they are sufficiently complete or not. This method predicts Type III, IV and VI secretion systems, which are all able to inject eukaryotic cells and to directly transfer proteins into the host cell cytosol. We used the support vector machine (SVM) approach from the recently extended PICA framework (18,19) to create a classification model for each secretion system. Genotypes are represented by COG/NOG presence profiles from the EggNOG 4.0 database (17). The prediction model was trained with COGs and NOGs of bacterial strains encoding the intact secretion system as positive samples (Supplementary Tables S1–S3). Due to the lack of available experimental data indicating non-intact secretion systems we have randomly sampled the negative dataset from all remaining relevant genomes in EggNOG 4.0, excluding all genera that contain positive samples (Supplementary Tables S1–S3). To maximize the predictive power of the secretion system models, we only considered genomes with clear indications for the intact nature of their secretion system, such as contained in SEED (20), SecReT (3,4) AtlasT4SS (21) and own searches in publications. We considered only complete genomes by requiring the existence of at least 39 of 40 phylogenetic marker genes (22). The SVM uses the default values suggested by PICA: type:C = 5, Kernel = linear, gamma = 0 (19). The whole workflow of EffectiveS346 is shown in Supplementary Figure S1. Technical documentation about the PICA models in EffectiveS346, lists of COGs and NOGs ranked by their relevance for the secretion systems, as well as lists of positive and negative training data are provided in the EffectiveDB web portal.

The input data for EffectiveS346 are genotype files listing COGs and NOGs according to EggNOG 4.0, which are present in a particular genome. Genotype files may optionally provide a mapping of the COGs/NOGs to protein names. To our knowledge no public web service is available for the assignment of EggNOG orthologous groups to proteins in user-provided genomes. Therefore, in order to facilitate the genotype prediction for user-provided genomes, the EffectiveDB web portal contains software modules for this purpose. They require protein sequences from nearly complete genomes as input. The initial gene prediction is intentionally left to the user, as the correct prediction of translation initiation sites is crucial for the detection of N-terminal signal peptides in other EffectiveDB methods. For any user-submitted FASTA file an optional check of genome completeness by CheckM (23) is provided. According to the prediction accuracy of PICA models for incomplete genomes we recommend that at a major fraction (default = 85%) of the marker genes should be present in order to obtain reliable secretion system predictions. The COGnitor program (24) predicts orthologous groups which serve as input for EffectiveS346. Alternatively, the user may submit own lists of EggNOG 4.0 COGs and NOGs present in a genome. In this case, the time-consuming homology search by COGnitor is omitted.

With the genotype list as input, the three SVM models calculate binary classifications whether the input species contains intact Type III, IV and VI secretion systems. These predictions feature a mean balanced accuracy of over 90% and a standard deviation of below 4.5% (Supplementary Tables S4–S7). Nevertheless, we showed that at least for the Types III and IV, in only 85% complete genomes more than 83% of the intact secretion systems could still be recognized. EffectiveS346 additionally provides lists of the 100 most important COGs in regard to each classification and lists of those COGs that are contained in the KEGG maps of the three secretion systems. From proteins of COGs, which are associated with the secretion systems in KEGG, EffectiveDB estimates the copy number of predicted secretion systems. These copy numbers are shown in the result summaries as well as in the detailed output files, combined with lists of the respective protein names. If protein names represent locus tags, these lists are useful to infer putatively intact (complete) clusters of secretion system genes and to their respective genomic regions. Among the 1677 pre-calculated bacterial genomes from EggNOG 4.0 we have predicted 164 intact Type III, 266 intact Type IV and 247 intact Type VI secretion systems. The Supplementary Figures S2–S7, created with Krona (25), visualize the taxonomic distribution of genomes with and without the three secretion systems. Genome contents and prediction overlaps with T346Hunter (2), SecReT4 (3) and SecReT6 (4) are given in Supplementary Figure S8 and Table S8, respectively.

### EffectiveT3, EffectiveCCBD and T4SEpre predict Type III and Type IV secreted proteins

The prediction of Type III secreted proteins in EffectiveDB was improved and extended. An updated version of EffectiveT3 (26) facilitates the recognition of N-terminal signal peptides. For the update we have assembled new training datasets, combining 504 verified secreted proteins from T3SEdb (7) along with our original training data (26). The new model is also a Naive Bayesian Classifier, trained with more data. Sequence similarity based elimination of redundancy, creation of features, selection of the most discriminating features, learning and testing procedure were performed as described initially (26). Training and classifying was performed with the Weka package (27). When performing a leave-one-out cross validation test, this yielded in an accuracy of 0.87 that is comparable to our previous report (26). In addition, a leave-one-taxon-out test was applied to prove that the model is still based on ubiquitous features of the signal and can thereby be applied to any taxon. In this test all proteins from one taxon are kept out from the training and are then exclusively used as test data. Overall, an average area under the curve (AUC) of 0.80 was obtained. The new model is now embedded into EffectiveDB and also available as EffectiveT3 module for download. A comparison between the performance of the old and new model, calculated as receiver operating curves, is shown in Supplementary Figure S9. All training data are provided on the website and in Supplementary Table S9. We run also BEAN 2.0 (8) on our new data. The results are similar and shown in Supplementary Figure S9 and Table S10. The default minimal score from the Naive Bayesian Classifier for the class ‘secreted’ is 0.9999 in the new model. This default value is called ‘selective’ at the webpage, whereas 0.95 is called ‘sensitive’. The threshold can also be freely chosen.

Complementing the signal peptide based prediction of Type III secreted proteins we have integrated a novel method for class IB chaperone prediction. Those chaperones facilitate the correct selection and unfolding of Type III dependent effector proteins (28). A commonly shared sequence within the region of the 70 N-terminal residues of Type III secreted proteins was shown to serve as binding site of the chaperones. This ‘conserved chaperone-binding domain’ (CCBD) follows the explicit pattern: (LMIF)_1_XXX(IV)_5_XX(IV)_8_X(N)_10_ (29). We implemented EffectiveCCBD, allowing to compare any given collection of protein sequences against this motif by using Biopython (30). The output informs the user whether the pattern was found within the expected region (26–70 amino acids from the N-terminus) or in the surrounding regions (1–25 and 71–150 amino acids from the N-terminus). Across the 1677 pre-calculated bacterial genomes we have observed fewer positive predictions by EffectiveCCBD (49 543) than by EffectiveT3 (361 189). For 8651 proteins both programs agree in their positive prediction, whereas 2 165 946 proteins are consistently predicted as not secreted.

For the prediction of Type IV secreted proteins we have integrated the program T4SEpre (12), which only requires amino acid sequences as input and is thus compatible with the other methods for secreted protein prediction in EffectiveDB. Due to the very high computational costs of the T4SEpre model based on protein secondary structure (Sse), we have only used the sequence-based models T4SEpre_psAac and T4SEpre_bpbAac. We used the published databases to calculate the amino acid properties and bi-residue properties with therein provided methods.

### EffectiveELD predicts secreted proteins based on eukaryotic-like domains, independently from the mode of transport

The implementation of EffectiveELD, which predicts secreted proteins based on ELD, was not changed since the initial version of EffectiveDB. However, besides the update of the genome repository and the protein domain database we have changed the presentation of ELD in the EffectiveDB web portal. Mean and standard deviation of the domain frequency in not host-associated genomes are now shown and can be exported into different file formats. This is mainly relevant for the analysis of proteins from metagenomic samples. Metagenome assembly artifacts may artificially increase the copy number of typically single-copy non-effector genes, such as as house-keeping genes. In these cases, the reported Z-score would indicate significant enrichment of such genes, which are certainly not effectors. This type of false positive matches can now be easily detected and excluded from further analysis.

In the ‘protein mode’ of EffectiveDB, analyzing arbitrary collections of protein sequences, only ELD with significant enrichment in at least one host-associated genome from the EffectiveDB genome repository are reported. In the new ‘genome mode’ of EffectiveDB, the Z-scores for the enrichment of ELD are automatically calculated *de novo* for all protein domains occurring in eukaryotic genomes. This allows the prediction of novel ELD that have not yet been observed in any of the host-associated genomes from the EffectiveDB genome repository.

### Predotar predicts subcellular targeting of effectors in the host cell

Multiple evidences suggest organelles as targets of bacterial secreted proteins (reviewed e.g. in 31 with regard to mitochondria). The program Predotar (32) is a tool that allows to rapidly screen N-terminal targeting sequences and to predict their subcellular localization in eukaryotic host cells. We have included this tool into EffectiveDB in order to facilitate downstream analysis of putative secreted proteins due to their predicted cellular location in the host. Predotar is used for both, the protein and the genome mode of EffectiveDB.

### EffectiveDB integrates predictions and Supplementary Data in an improved website

**Figure 2.**
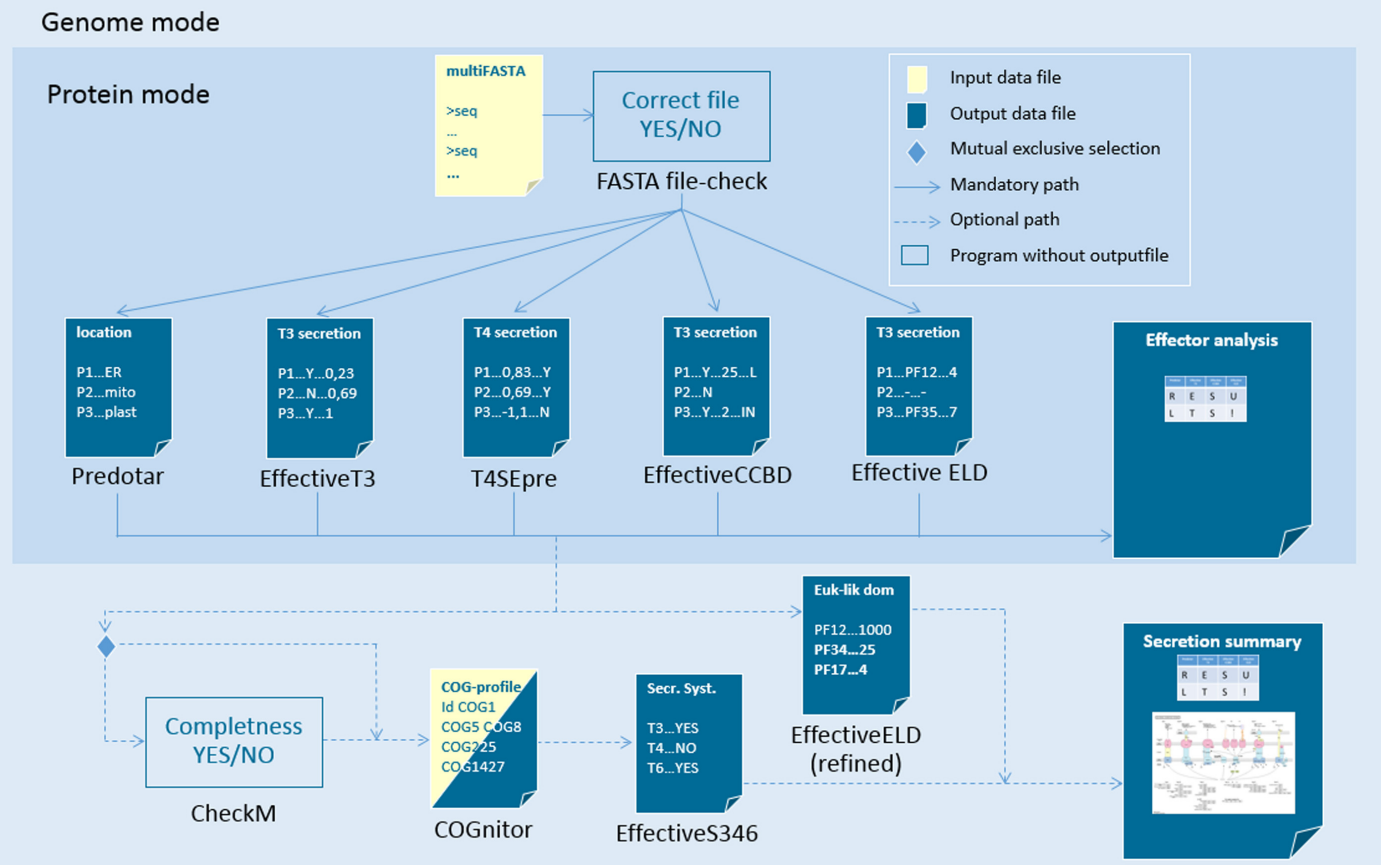
Schematic output of EffectiveDB. The EffectiveDB output consists of predictions for individual proteins and for the entirety of proteins in a complete genome. Protein-based predictions (Type III secretion peptides, Type III chaperone binding sites, Type IV secretion peptides, eukaryotic-like domains and subcellular targeting) are grouped. Genome-based results are provided as summary and as lists of most relevant orthologous groups for each secretion system.

A new website has been implemented for EffectiveDB. It provides interactive access to the pre-calculated predictions of secreted proteins and secretion systems. A submission form is provided to specify input data and parameters for EffectiveDB calculations of user-submitted data. No data from these interactive calculations are stored in EffectiveDB. Results are only provided to the owner of a submission and are deleted after one month. All predictions are presented to the user in an integrative manner: equivalent predictions, such as all predictions based on an individual protein sequence, are grouped (Figure 2). For the genome-based predictions by EffectiveS346 the web site provides a summary as well as the detailed lists of the most relevant proteins for each secretion system.

## OUTLOOK

We will continue to update EffectiveDB on a yearly basis. In consideration of promising current research in other teams we expect that further signal peptide based predictions of type IV secreted proteins can be added in the next release. The pre-calculation of secretion systems in complete genomes is linked to the EggNOG database, which provides the genotype lists. Any future update of EggNOG will therefore also trigger an update and significant extension of EffectiveDB. We will continue to incorporate additional bacterial genomes from NCBI RefSeq, which are not yet contained in EggNOG.

## SUPPLEMENTARY DATA

Supplementary Data are available at NAR Online.
